# Laser-induced topological spin switching at room temperature in the van der Waals ferromagnet Fe_3_GaTe_2_

**DOI:** 10.1038/s44306-026-00139-x

**Published:** 2026-06-01

**Authors:** Charlie W. F. Freeman, Woohyun Cho, Paul S. Keatley, PeiYu Cai, Zekun Xue, Chenghao Yang, Harry Youel, Elton J. G. Santos, Robert J. Hicken, Heejun Yang, Hidekazu Kurebayashi, Murat Cubukcu, Maciej Dąbrowski

**Affiliations:** 1 London Centre for Nanotechnology, University College London, London, UK; 2 National Physical Laboratory, Teddington, UK; 3 Department of Electronic and Electrical Engineering, University College London, London, UK; 4 Department of Physics, Korea Advanced Institute of Science and Technology (KAIST), Daejeon, Republic of Korea; 5 Department of Physics and Astronomy, University of Exeter, Exeter, UK; 6 School of Physics and Astronomy, The University of Edinburgh, Edinburgh, UK; 7 Department of Physics and Astronomy, University College London, London, UK; 8 Donostia International Physics Center (DIPC), Donostia-San Sebastián, Spain; 9 WPI-AIMR, Tohoku University, Katahira, Japan; 10 Center for Science and Innovation in Spintronics, Tohoku University, Sendai, Japan; 11 Institute for Materials Research, Tohoku University, Sendai, Japan

**Keywords:** Materials science, Physics

## Abstract

We demonstrate room-temperature nucleation and manipulation of topological spin textures in the van der Waals (vdW) ferromagnet Fe_3_GaTe_2_ using laser-pulse excitation. Rapid laser-induced heating followed by cooling enables access to the skyrmion bubble state at low fields and drives reversible switching between this state and labyrinth domains. The switching requires a minimum of about 20 pulses, and further reduction of the pulse number is limited by sample degradation at higher fluence. The nucleation occurs at magnetic induction fields as low as 5 mT, which substantially lowers the field requirement compared to slow field-cooling approaches. Micromagnetic simulations attribute this switching to the thermal cycle induced by the laser. Our findings establish vdW ferromagnets as promising candidates for room-temperature, laser-controlled, non-volatile memory storage applications.

## Introduction

The discovery of stable, long-range magnetic order in single-layer vdW materials has stimulated a new interest in the study of layered magnetic materials, particularly for future spintronic applications^1–7^. Most known vdW ferromagnets host a rich variety of topological spin textures in their pristine state. However, the microscopic mechanisms underlying their formation, and in particular the origin of the Dzyaloshinskii-Moriya interaction (DMI), remain an active topic of discussion, as many of these materials possess global inversion symmetry. Studies of Fe_3_GeTe_2_, one of the most thoroughly investigated two-dimensional (2D) magnets, have shown that topological spin textures can arise from several mechanisms, including intrinsic vacancy-induced inversion-symmetry breaking leading to DMI^8^, higher-order interactions^9^, and the interplay between dipolar interactions and strongly temperature-dependent out-of-plane magnetic anisotropy^10^. For both Fe_3_GeTe_2_ and Cr_2_Ge_2_Te_6_, field-cooling procedures (i.e. cooling under an applied magnetic field) have proven particularly effective in inducing and stabilising metastable skyrmions at temperatures where they do not form under conventional field-sweep protocols^8,10–12^. More recently, laser pulses have emerged as a powerful tool for creating and manipulating topological spin textures^13–16^. Through ultrafast demagnetisation^17^, laser excitation enables rapid heating and subsequent cooling, generating strongly spatially inhomogeneous thermal profiles and driving the system into non-trivial topological states that are inaccessible via standard field-cooling methods^16,18^. Unlike conventional field- cooling, which is a slow, near-equilibrium process that follows the magnetic phase diagram, laser-induced heating and cooling proceed through a spatially inhomogeneous and nonadiabatic pathway, in particular in the case of high-fluence laser pulses, allowing the system to evolve into metastable topological states beyond equilibrium phase boundaries. Ultrashort laser pulses therefore provide both unprecedented temporal resolution for studying the transformation of spin textures and a fast, potentially energy-efficient route for their manipulation, while enabling access to metastable states that cannot be obtained using quasi-equilibrium approaches. For potential applications, deterministic toggle switching between states is essential, similar to all-optical switching (AOS) of uniform/monodomain magnetisation^19–21^. While vdW magnets have demonstrated AOS^22,23^ and optical control of topological textures^18^, such studies have so far been restricted to cryogenic temperatures due to their Curie temperatures being below room temperature.

The synthesis of Fe_3_GaTe_2_ represents an important advancement toward vdW systems operating beyond room temperature, with a Curie temperature (*T*_C_) of 350 –370 K, thereby enabling the exploration of spintronic applications for vdW magnets under ambient conditions^24–28^. Spin structures such as skyrmions, antiskyrmions, and skyrmion bags have already been reported for Fe_3_GaTe_2_, including demonstrations of zero-field skyrmion stability from temperatures above room temperature down to 100 K, when formed via field-cooling procedures^25,29–32^. Owing to the significant contribution of dipolar interactions to the stabilisation of skyrmions in Fe_3_GaTe_2_, their size and density can be efficiently tuned via film thickness and applied magnetic field^25,30^. Current-driven skyrmion motion has been observed at room temperature using electrical current densities on the order of 10^9^ A/m^2^, which is approximately two orders of magnitude lower than those required in other van der Waals magnets^33^. Further studies have demonstrated the manipulation and formation of skyrmions through field-cooling protocols combined with stray fields from magnetic force microscopy tips^34^, as well as skyrmion nucleation via ultrafast laser writing in non-stoichiometric Fe_2.84±0.05_GaTe_2_^29^, where ultrafast heating followed by quenching in an external magnetic field leads to the formation of skyrmion spin textures. These unique properties of Fe_3_GaTe_2_, together with its exceptional thermal stability of skyrmions up to 355 K, make this material a highly promising platform for hosting topological spin textures and for future spintronic applications.

In this study, we demonstrate the nucleation of topological spin textures at room temperature and under low magnetic fields using laser pulses. As we cannot directly resolve skyrmions or identify the topological charge *Q* due to the limited resolution of our optical method, we will refer to the skyrmion bubbles (type-I bubbles) phase whenever we discuss circular domains with magnetisation antiparallel to the surrounding out-of-plane spin orientations. We assume that the skyrmion bubbles observed in our experiments possess non-vanishing topological charge, in contrast to topologically trivial bubbles with *Q* = 0 (type-II bubbles). This assumption is justified, as several independent experiments have already confirmed that Fe_3_GaTe_2_ hosts skyrmions and skyrmion bubbles across various temperatures and thicknesses^25,29–32,35,36^. The laser excitation induces ultrafast heating of the magnetic system, which quenches the magnetic order. In the presence of an external magnetic field, this process enables access to the skyrmion bubble phase. We also show that it is possible to reversibly switch between two distinct spin textures, namely the skyrmion bubble state and the labyrinth domain state, by applying specific combinations of laser and magnetic field protocols. This broadens the strategies available for controlling topological spin textures through laser manipulation. Micromagnetic simulations further confirm that laser-driven heating followed by cooling stabilises different magnetic phases depending on the applied field.

## Results

### Kerr microscopy measurements

**Fig. 1:**
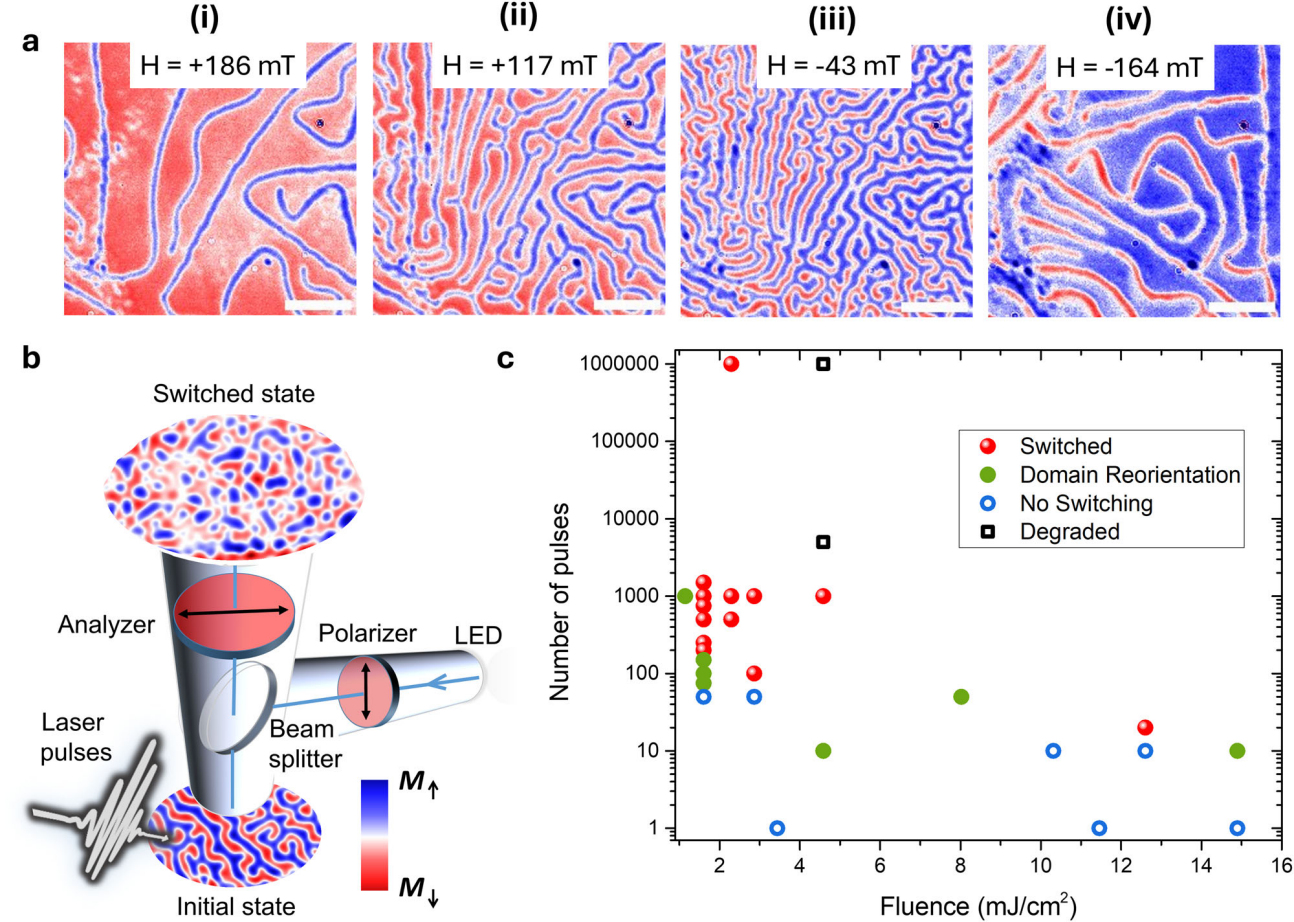
Magnetisation reversal and laser-induced topological spin switching. **a** Wide-field Kerr microscopy (WFKM) images acquired at applied magnetic fields of (i) 186 mT, (ii) 117 mT, (iii) −43 mT, and (iv) −164 mT, showing magnetisation reversal. The scale bar is 5 μm. **b** Schematic of the experimental setup for WFKM combined with external laser excitation. WFKM images show laser-induced switching at remanence from the initial labyrinth state to a skyrmion bubble structure with optical pumping with 10^6^ pulses at 2.3 mJ/cm^2^. **c** Diagram showing the switching behaviour as a function of fluence and the number of pulses. The measurements were performed at room temperature.

Fe_3_GaTe_2_ has a hexagonal structure with space group P6_3_/mmc with the crystalline axes defined by *a* = *b* = 3.9860 Å, *c* = 16.2290 Å, *α* = *β* = 90°, *γ* = 120°^24^. In its bulk single crystal, Fe_3_GaTe_2_ has a saturation magnetisation (*M*_*s*_) of 40.11 emu/g and large perpendicular magnetic anisotropy, *K*_U_, of −4.79 × 10^5 ^J/m^3^^24^. We employ wide-field Kerr microscopy (WFKM) in polar geometry to probe the domain structure, the magnetisation reversal, and optically-induced topological spin textures. Bulk-like flakes, approximately 50 μm thick with lateral dimensions of around 1 mm^2^, were prepared by cleaving the Fe_3_GaTe_2_ crystal mounted on the WFKM sample holder using Blue Nitto tape. In Fig. 1a, we present representative WFKM images at selected magnetic field values. The saturation field at room temperature is found to be approximately 280 mT. The observed hysteresis loop is typical of materials with perpendicular magnetic anisotropy that undergo nucleation of labyrinth (or alternatively called stripe) domains as the field decreases (see Supplementary Fig. S1). The average width of the stripe domain is found to be 370 ± 90 nm through ridge detection analysis at zero field and room temperature^37^.

Changes to the magnetic state after optical pumping were recorded by imaging the initial (before) and final (after excitation) states, following excitation with 1 MHz repetition rate laser pulses of photon energy 1.2 eV with a 300 fs pulse duration, using variable fluence and pulse numbers. Figure 1b shows the experimental setup, where the initial remanent labyrinth domain state is switched to a skyrmion bubble domain final state through the application of 10^6^ laser pulses. This remanent state is not truly a zero-field condition due to a residual field from the electromagnet, estimated to be approximately 5 mT. In Fig. 1c, a diagram of pump fluence versus the number of pulses required for switching or domain reorientation is shown. These measurements show that switching can be achieved over a broad range of fluences and pulse numbers, where reducing the pulse fluence requires a corresponding increase in the number of pulses to reach the switched state. The minimum number of pulses required for switching was 20 at a fluence of 12.6 mJ/cm^2^, while the lowest fluence at which switching was observed was 1.6 mJ/cm^2^ when using 200 pulses. The observation of switching and domain reorientation using multiple pulses points to a heat accumulation effect. We also performed pumping at increased fluence, above 15 mJ/cm^2^, to achieve single-shot switching. This leads to degradation of the sample (see exemplary images in Supplementary Fig. S2), which is generally expected for GaTe-based vdW compounds under ambient conditions^38^, especially when considering such high laser fluence. To overcome this, further material optimisation, such as encapsulation and pumping with different wavelengths can be undertaken to enable single-shot switching. In addition, the laser-induced heating is expected to be more efficient in thin flakes due to the domain memory effect^39^, which is associated with the low thermal conductivity of vdW layers. As a result, magnetic moments lying well below the penetration depth of the optical pump remain intact and help restore the magnetic order in the upper, demagnetised layers. However, as the domain size decreases with thickness, we were unable to confirm the effect of thickness on the formation of topological spin textures due to the limited resolution of our optical methods (see Supplementary Fig. S3 for studies on a thin ~ 20 nm flake). To verify this thickness dependence, higher-resolution techniques, such as Lorentz transmission electron microscopy^28^, are required.

**Fig. 2:**
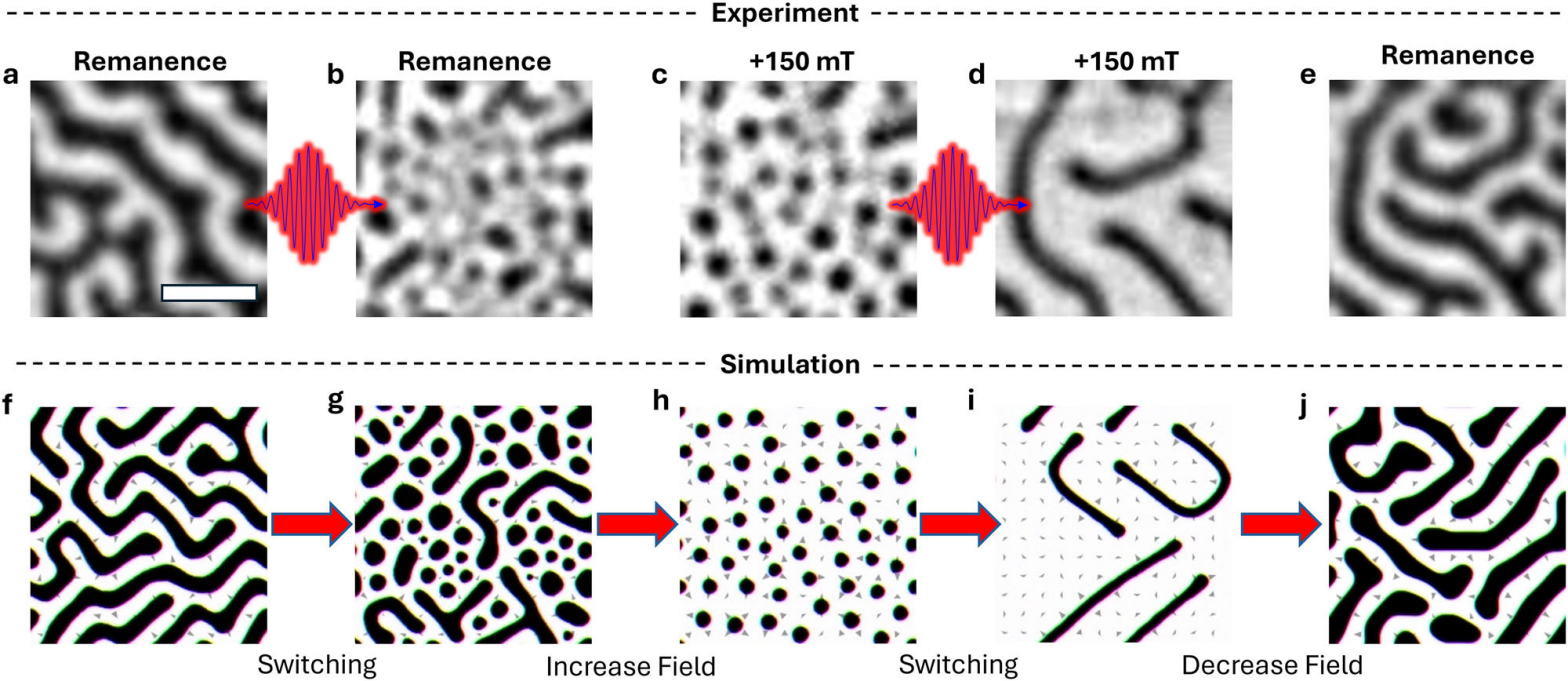
Spin toggle switching. **a**–**e** Light-induced toggle switching between labyrinth and skyrmion bubble states observed via WFKM. Starting from an initial remanent labyrinth domain structure (**a**), 10^6^ laser pulses at 2.3 mJ/cm^2^ induce a transition to a magnetic configuration dominated by the skyrmion bubble state (**b**), which becomes even more clearly identifiable after applying a magnetic field of +150 mT (**c**). Subsequent exposure of the skyrmion bubble state to laser pulses triggers another topological spin switching, leading to a transition back to the labyrinth state (**d**). Removal of the magnetic field (**e**) restores the system to the same state as in (**a**). The scale bar has length 2 μm. **f**–**j** Micromagnetic simulations replicate the sequence shown (**a**–**e**). The simulation size is 1 × 1 μm × 50 nm, with switching achieved under fields of +11 mT (**f**, **g**) and +140 mT (**h**, **i**).

We now demonstrate the protocol used for toggle switching between two distinct states, depicted in Fig. 2. Initially, the labyrinth state is prepared by sweeping the magnetic field from saturation to remanence. The laser is then applied with a remanent field (~5 mT), resulting in a transition to the skyrmion bubble state. Subsequently, an out-of-plane magnetic field of +150 mT is applied, leading to a more distinct formation of skyrmion bubbles, with the average domain area decreasing from 0.3 to 0.2 μm^2^ (Fig. 2c), which are close to the resolution limit of the WFKM. Based on previous reports^25,28,29,36^, smaller skyrmion bubbles are likely present in the system but cannot be resolved with our method. By applying laser pulses again to the skyrmion bubble state at +150 mT, we induce a transition back to the labyrinth state (Fig. 2d). Finally, upon removal of the magnetic field, the remanent labyrinth state is recovered, characterised by equally sized and populated up and down domains (Fig. 2e). Notably, in contrast to non-stoichiometric Fe_2.84±0.05_GaTe_2_^29^, where one-way switching to skyrmions required an external field, we demonstrate two distinct switching events: from the labyrinth state to skyrmion bubbles and back to the labyrinth state, via optical pumping at remanence and under an external field, respectively.

### Micromagnetic simulations

To gain a deeper understanding of the mechanism behind this switching behaviour, we performed micromagnetic simulations using MuMax3^40^. Using representative values from the literature for the exchange stiffness *A*_ex_, magnetic anisotropy energy, saturation magnetisation, and DMI, we simulate a geometry of 1 μm × 1 μm × 50 nm ^28^ (see ‘Methods’ for details). To reproduce the laser-induced heating, the system was evolved for 20 ns at fixed temperature steps between 300 and 370 K. All images of the domain structure from the simulations were extracted after cooling down to the base temperature of 300 K. We closely reproduce the experimental data by applying conditions similar to the experimental process, though the exact agreement with the applied fields is not achieved, and attributed to limitations in the system size in the simulation (Fig. 2f–j) as the real bulk thickness of 50 μm is too large to simulate. The field values chosen here provide the closest match to the experimental data.

**Fig. 3:**
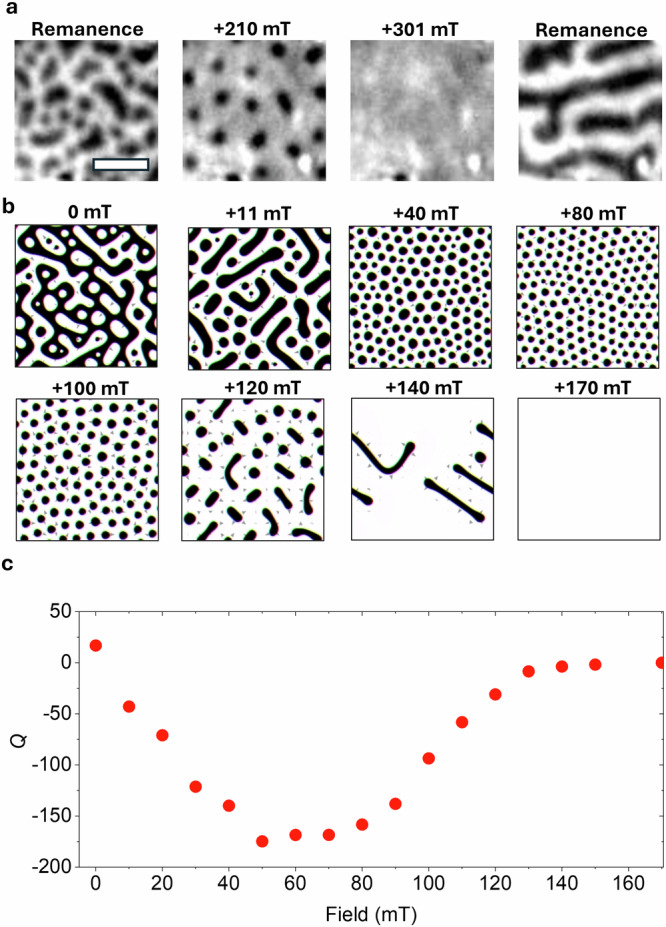
Magnetic field stability of nucleated skyrmion bubble domains. **a** The effect of the applied magnetic field on the laser-induced switched state obtained at remanence after optical pumping for 1500 pulses at 1.6 mJ/cm^2^. The scale bar is 2 μm. **b** Micromagnetic simulations replicating laser-induced heating under different magnetic field values. The simulation size is 1 μm × 1 μm × 50 nm. **c** Final topological charge as a function of applied field calculated from the simulations in (**b**).

We now demonstrate the field stability of the nucleated skyrmion bubble domains. In Fig. 3a, it can be seen that upon increasing the field, the skyrmion bubble domains remain stable up to magnetic fields just below the saturation field. When the field is increased beyond saturation and subsequently reduced back to remanence, the labyrinth domains are recovered. In Fig. 3b, we present simulation results for various applied field values during which the heating process was applied, in order to replicate laser-induced changes to the magnetic structures. At zero field, a hybrid state of up and down bubbles/stripes is observed, similar to previous results reported in Cr_2_Ge_2_Te_6_^18^. Hybrid skyrmion/stripe states have also been observed in Fe_3_GaTe_2_^25,29^, and their presence is associated with the competition between dipolar interactions and DMI. The application of a small positive field breaks the symmetry of the system, promoting the nucleation of skyrmion bubbles with a uniform polarity. However, at low fields (+11 mT), there is significant size variation, with many elongated bubbles being stabilised. As the field increases, the number of skyrmion bubbles domains increases, although notable size variation persists relative to an ideal skyrmion lattice. This is attributed to the role of dipole-dipole interactions competing with DMI at low fields when stabilsing skyrmion bubble domains^30^. When a field of 120 mT is applied during laser-induced heating, the skyrmion bubble domains are more sparsely nucleated, and many domains exhibit elongation. At an applied field of 140 mT, only labyrinth domains are observed after the laser pulse, indicating that a large field beyond the skyrmion bubble formation window is required to generate labyrinthine/stripe domains. Finally, at fields above a saturation only a uniform domain state is observed. The final topological charge is then calculated and shown in Fig. 3c. Here, the topological charge, *Q*, is defined as

1 $$Q=\frac{1}{4\pi }\int {\bf{n}}\cdot \left(\frac{\partial {\bf{n}}}{\partial x}\times \frac{\partial {\bf{n}}}{\partial y}\right){d}^{2}{\bf{r}}$$

where **n** is the direction vector of magnetisation, and a skyrmion has a topological charge of −1^41^. The highest number of skyrmion bubbles (minimum *Q*) is achieved at an applied field of 50 mT. Beyond this field the topological charge gradually increases to zero, reflecting the reduced skyrmion bubble density and the re-emergence of labyrinth domains before the uniform domain above the saturation field. This behaviour demonstrates that the number of skyrmion bubbles can be finely tuned via the applied magnetic field during the switching process.

## Discussion

Previous studies employing slow field-cooling procedures on Fe_3_GaTe_2_^28,34,42^ have shown the stabilisation of skyrmion bubble domains within a field window bounded between 32 and 55 mT. In this study, we demonstrate skyrmion bubble nucleation at approximately 5 mT, representing a five-fold reduction in the applied field required. The observed switching can be understood as a process driven by laser-induced heating followed by subsequent cooling, with the applied field determining the stabilised domain state. When the laser fluence is sufficient to heat the sample above *T*_C_, the metastable state is erased, and a new domain configuration emerges during the cooling phase after the laser pulse. Although the precise timescale of the heating-cooling cycle remains uncertain and requires further time-resolved experiments, the process substantially reduces the field required for skyrmion bubble domain formation compared to conventional slow field-cooling in literature, consistent with recent reports on laser-induced skyrmion bubbles^43,44^.

Optical manipulation of topological spin structures offers significant advantages over traditional approaches, enabling ultrafast generation and annihilation of skyrmions on picosecond timescales and providing additional parameter space for controlling their size and density^15^. Our results also highlight technical limitations relevant to potential applications, most notably that the higher laser fluence required to reduce the number of excitation pulses can lead to sample degradation due to heat accumulation. This challenge may be mitigated by using thinner flakes, optimising heat dissipation into the underlying substrate^39^, or through encapsulation. Such approaches, however, would require alternative experimental techniques for detection, as the reduced domain and spin-texture sizes in thinner samples fall below the diffraction-limited resolution of optical methods. Further developments may include local excitation and manipulation using tightly focused laser beams, enabling selective control of individual spin textures, as recently demonstrated in W/CoFeB systems with high-precision trapping and transport of single skyrmions^45^. Finally, the use of high laser fluence to generate strong nonequilibrium fluctuations^44^ may provide access to additional metastable states that are not readily achievable using conventional approaches.

In summary, we have demonstrated reversible, laser-driven toggle switching between labyrinth and skyrmion bubble domain states at room temperature in the vdW ferromagnet Fe_3_GaTe_2_. The switching is enabled by laser-induced heating and rapid quenching of the magnetic order, a mechanism further corroborated by micromagnetic simulations. These findings establish a versatile route for optical control of spin textures in vdW magnets and highlight the potential of such control for ultrafast, non-volatile memory technologies.

## Methods

### Material growth

Fe_3_GaTe_2_ crystals were synthesised by a self-flux method to preclude any possibility of extrinsic doping and contamination. High-purity Fe powder (99.9%, Thermo-fisher scientific) and Te powder (99.999%, 5N-plus) were ground in a stoichiometric ratio and placed in the quartz tube with Ga granules (99.99999%, Alfa-aesar). The quartz tube was sealed under a high vacuum (<1 × 10^−5^ hPa) and heated up to 1373 K within 5 h. The temperature was held for 30 h to ensure the melting of mixtures, and the tube was rapidly cooled down to 1103 K. After the cooldown, the temperature was maintained over 150 h, and the quartz tube was quenched in air. Single crystals were then separated from the residual flux by mechanical cleaving and exfoliation with Scotch tape so that plate-shaped crystals with typical size of 5 × 5 × 0.1 mm^3^ were typically obtained.

### Wide-field Kerr microscopy (WFKM)

The polar Kerr effect was employed to probe out-of-plane magnetisation under magnetic-field or optical-pulse excitation. Linearly polarised light illuminated the sample, and polarisation changes in the reflected beam were detected as intensity variations using a nearly crossed analyser, a quarter-wave plate, and a high-sensitivity CMOS camera. For optically induced magnetisation changes, the fundamental laser output at 1035 nm with a 300 fs pulse duration was used as an optical pump, with p-polarised light at a 1 MHz repetition rate incident at 45° to the sample plane and focused to a 90 μm diameter spot (intensity at 1/*e*^2^)^21^. The number of pulses was controlled via an external trigger signal (a TTL pulse of specified width) generated by a function generator. All measurements were conducted at room temperature. All images presented in this work are acquired for the final magnetisation state which remains the same until the sample is exposed either to more optical pulses, or to an external magnetic field.

### Micromagnetic simulations

Micromagnetic simulations were performed using MuMax3^40^. A simulation grid of 200 × 200 × 10 cells with a cell size of 5 × 5 × 5 nm^3^ was used, along with periodic boundary conditions (10,10,0) Magnetic parameters were scaled phenomenologically according to [*M*_*s*_(*T*)/*M*_*s*_(300 K)] = ${[{K}_{u}(T)/{K}_{u}(300K)]}^{1/3}$ = ${[{A}_{ex}(T)/{A}_{ex}(300K)]}^{1/2}$ = [*D*(*T*)/*D*(300 K)]^1/2^^46,47^, where *M*_*s*_ (300 K) = 3.76 × 10^−5 ^A/m, *K*_*u*_ (300 K) = 3 × 10^5 ^J/m^3^, *A*_*ex*_ (300 K) = 7.5 × 10^−12^ J/m, *D* (300 K) = 1.6 × 10^−3 ^J/m^2^. We assume that *M*_s_(*T*) varies linearly in this temperature region; however, further improvements could be made by using experimental *M*_s_(*T*) data. Magnetic parameters were taken from the literature, with the DMI being chosen for good visual match to experimental data^24,28,29^. The damping constant was set to 0.8 to reduce simulation time. The initial labyrinth domain state was prepared by zero-field cooling from a randomised magnetisation configuration, followed by energy minimisation. To emulate laser-induced heating, the system was evolved for 20 ns at selected temperatures between 300 and 370 K. Temperature effects were incorporated by scaling the magnetic parameters according to the corresponding temperature.

## Supplementary information

Supplementary Information

## Data Availability

The datasets generated during the current study are available in the UCL Research Data Repository 10.5522/04/31231996.
