# Supplementary material for: Laser-induced topological spin switching at room temperature in the van der Waals ferromagnet Fe3GaTe2
Source: Npj Spintron. 2026 Jun 1;4(1):24. doi: 10.1038/s44306-026-00139-x (PMC13226038; doi:10.1038/s44306-026-00139-x)
Supplement: Supplementary file 1 — Supplementary Information [file 44306_2026_139_MOESM1_ESM.pdf]

Supplementary Information for:

Laser-induced topological spin switching at room temperature in the van der Waals ferromagnet Fe_3GaTe_2

Charlie W. F. Freeman[1,2,3†], Woohyun Cho[4], Paul S. Keatley[5], PeiYu Cai[6], Zekun Xue[1,3], Cheng-hao Yang[1,3], Harry Youel[1], Elton J. G. Santos[6,7], Robert J. Hicken[5], Heejun Yang[4], Hidekazu Kurebayashi[1,3,8], Murat Cubukcu[1,2†], Maciej Dąbrowski[5]†

[1]*London Centre for Nanotechnology, University College London, London, WC1H 0AH, UK*

[2]*National Physical Laboratory, Teddington, TW11 0LW, UK*

[3]*Department of Electronic and Electrical Engineering, University College London, London, WC1E 7JE, UK*

[4]*Department of Physics, Korea Advanced Institute of Science and Technology (KAIST), Daejeon 34141, Republic of Korea*

[5]*Department of Physics and Astronomy, University of Exeter, EX4 4QL, Exeter, UK*

[6]*School of Physics and Astronomy, The University of Edinburgh, Edinburgh EH9 3FD, UK*

[7]*Donostia International Physics Center (DIPC), 20018 Donostia-San Sebastián, Spain*

[8]*WPI-AIMR, Tohoku University, 2-1-1, Katahira, Sendai 980-8577, Japan*

[†]*Corresponding authors: uceecwf@ucl.ac.uk, m.cubukcu@ucl.ac.uk, m.k.dabrowski@exeter.ac.uk*

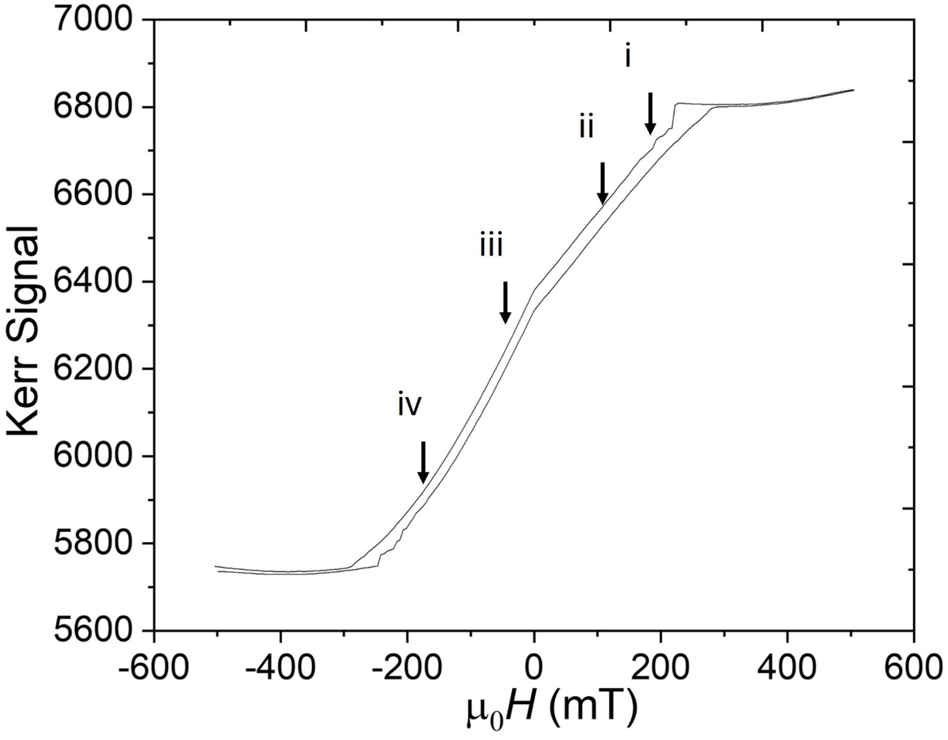

Figure S1: Hysteresis loop of the bulk sample used in the main text, extracted from the WFKM images. The labels i - iv correspond to the images at the fields shown in Fig. 1(a) of the manuscript.

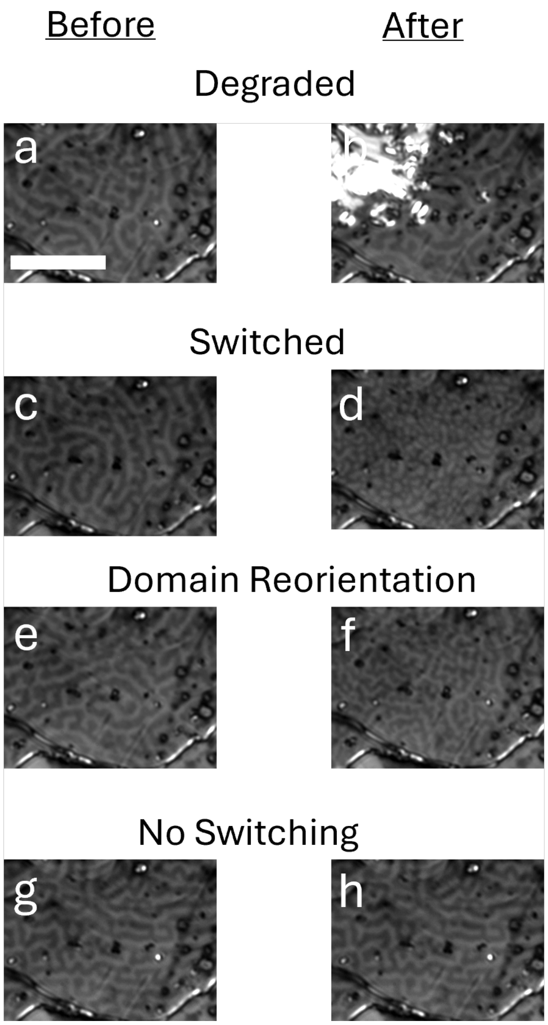

Figure S2: Examples of each region in the pulse number-power diagram in Fig. 1(d) of the main text. Left column is the remanent labyrinth state prepared by sweeping field from saturation to remanence, right column is the result after the application of laser pulses.(a-b) Degradation is observed at a single pulse of 20 mJ/cm^2 and at 5 mJ/cm^2 for 5000 pulses or more, resulting in a permanent change to the sample. (c-d) Switching is observed with the formation of the skyrmion bubble domains. (e-f) Domain reorientation is defined as a change in the domain size and shape while still retaining substantial fraction of domains in the labyrinth state. (g-h) No switching observed occurs at pulses numbers below 20 and powers below 1.6 mJ/cm^2. The scale bar is 10 μm.

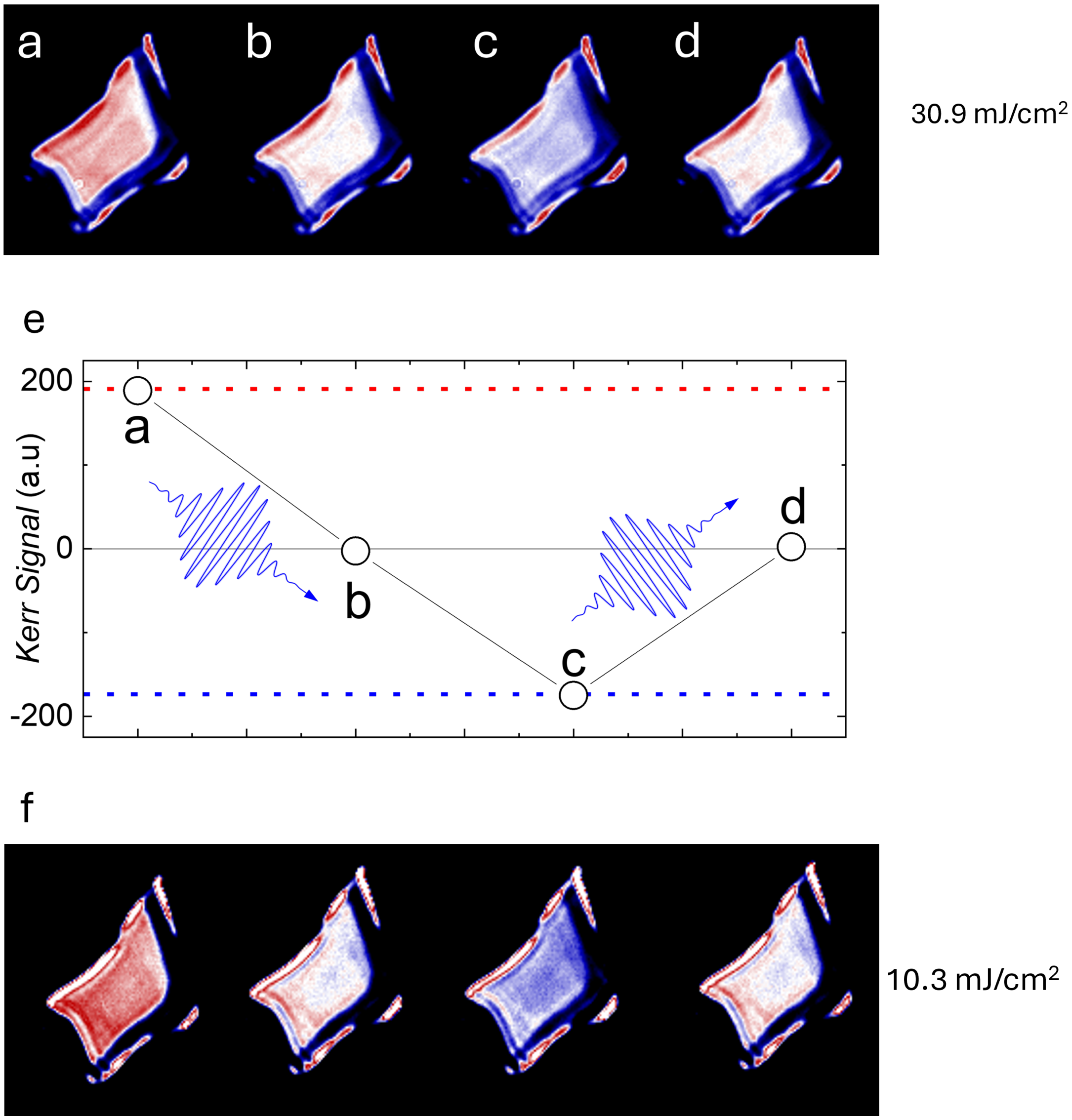

Figure S3: (a-e) WFKM images of ∼20nm thick Fe_3GaTe_2 exfoliated flake before (a,c) and after (b,d) application of 10^6 pulses at 30.9 mJ/cm^2, from positive remanence (red) and negative remanence (blue) respectively. (e) Plot of spatially averaged Kerr signal for a sample from (a-e), it is seen that the application of laser pulses demagnetises the sample from its remanent state, the domains formed are too small to resolve in the setup. (f) Same sequence as (a-e) with the lowest switching power (10.3 mJ/cm^2 at 10^6 pulses) to observe demagnetisation of the flake.
